# Dosimetric benefit to organs at risk following margin reductions in nasopharyngeal carcinoma treated with intensity-modulated radiation therapy

**DOI:** 10.1186/s40880-015-0016-8

**Published:** 2015-05-20

**Authors:** Yan-Ping Mao, Wen-Jing Yin, Rui Guo, Guang-Shun Zhang, Jian-Lan Fang, Feng Chi, Zhen-Yu Qi, Meng-Zhong Liu, Jun Ma, Ying Sun

**Affiliations:** Department of Radiation Oncology, Sun Yat-sen University Cancer Center; State Key Laboratory of Oncology in South China; Collaborative Innovation Center for Cancer Medicine, Guangzhou, Guangdong 510060 Peoples Republic of China; Department of Radiation Oncology, Cancer Center of Guangzhou Medical University, Guangzhou, Guangdong 510095 Peoples Republic of China

**Keywords:** Margin, Dosimetry, Organs at risk, Cone beam computer tomography, Nasopharyngeal carcinoma

## Abstract

**Introduction:**

It is important to decrease the radiation exposure of normal tissue in intensity-modulated radiation therapy (IMRT). Minimizing planning target volume (PTV) margins with more precise target localization techniques can achieve this goal. This study aimed to quantify the extent to which organs at risk (OARs) are spared when using reduced margins in the treatment of nasopharyngeal carcinoma (NPC).

**Methods:**

Two IMRT plans were regenerated for 40 patients with NPC based on two PTV margins, which were reduced or unchanged following cone beam computed tomography online correction. The reduced-margin plan was optimized based on maximal dose reduction to OARs without compromising target coverage. Dosimetric comparisons were evaluated in terms of target coverage and OAR sparing.

**Results:**

Improvements in target coverage occurred with margin reduction, and significant improvements in dosimetric parameters were observed for all OARs (*P* < 0.05) except for the right optic nerve, chiasm, and lens. Doses to OARs decreased at a rate of 1.5% to 7.7%. Sparing of the left parotid and right parotid, where the mean dose (D_mean_) decreased at a rate of 7.1% and 7.7%, respectively, was greater than the sparing of other OARs.

**Conclusions:**

Significant improvements in OAR sparing were observed with margin reduction, in addition to improvement in target coverage. The parotids benefited most from the online imaging-guided approach.

## Background

Nasopharyngeal carcinoma (NPC) is endemic in particular regions such as Southeast Asia. The annual incidence varies from 15 to 50 per 100,000 people in South China [1]. Radiation therapy is the primary approach for treating locoregionally confined NPC.

Intensity-modulated radiation therapy (IMRT) provides excellent locoregional control and sparing of organs at risk (OARs) in NPC [2-4] and has gradually replaced two-dimensional conventional radiotherapy as the first-line radiotherapy technique. Although the sparing of OARs has improved significantly with IMRT, late toxicities such as grades 2 to 4 xerostomia and sensorineural hearing loss still occur with incidences of 39.3% and 37.0%, respectively [4,5]. Therefore, improvements in OAR sparing and reductions in radiation toxicity remain to be important issues. Minimizing planning target volume (PTV) margins with more precise target localization techniques decreases the dose delivered to OARs [6-8]. However, little data have quantified the extent to which OARs are spared by reducing margins in NPC radiotherapy up to date.

Safety margins can be reduced with many imaging-guided radiation therapy technologies. By providing better resolution and three-dimensional (3D) images, conebeam computed tomography (CBCT) allows for a more accurate verification of the pretreatment position. Furthermore, the combination of CBCT imaging equipment, treatment couch, and automatic software for online correction allows for real-time monitoring and accurate online corrections [6-10]. Current literature has reported that CBCT online corrections can shrink safety margins in head and neck cancer by approximately 50% [11,12]. Quantification of the potential dosimetric benefit from margin reductions with CBCT online correction in NPC would be helpful to evaluate the value of CBCT.

Therefore, we generated two radiotherapy plans with pre-correction and post-correction PTV margins obtained through CBCT online correction. We then quantified the extent of OAR sparing with margin reductions without compromising PTV coverage. Additionally, we examined whether the dosimetric benefits observed from margin reductions using CBCT were associated with tumor stage and volume.

## Patients and Methods

### Patient characteristics

Between October 2010 and October 2011, 40 consecutive patients with newly diagnosed, untreated, and non-disseminated NPC were retrospectively included in the study. Approval for retrospective analysis of the patient data was obtained from the ethics committee of Sun Yat-sen University Cancer Center. Written consent was waived, and oral consent from the patients was obtained via telephone and documented by telephone recording. The use of oral consent was approved by the Institutional Review Board.

All patients underwent a pre-treatment evaluation, which included a complete medical history, physical and neurological examinations, hematology and biochemistry profiles, magnetic resonance imaging (MRI) scans of the neck and nasopharynx, chest radiography, and abdominal ultrasonography. Patients with N2 or N3 lesions underwent emission computed tomography (ECT) or positron emission tomography-computed topography (PET-CT). Medical records and images were analyzed retrospectively, and all cases were staged according to the 7th edition American Joint Committee on Cancer (AJCC) staging system. Clinical characteristics are listed in Table 1.

**Table 1:** **Clinical characteristics of the 40 patients with nasopharyngeal carcinoma (NPC)**

**Characteristic**	**No. of patients (%)**
Age (years)	
Median	46
Range	21–63
Sex	
Male	32 (80.0)
Female	8 (20.0)
Histology	
WHO I	0 (0)
WHO II/III	40 (100.0)
T category^a^	
T1	7 (17.5)
T2	9 (22.5)
T3	15 (37.5)
T4	9 (22.5)
N category^a^	
N0	10 (25.0)
N1	23 (57.5)
N2	6 (15.0)
N3	1 (2.5)
Clinical stage^a^	
I	5 (12.5)
II	10 (25.0)
III	15 (37.5)
IV	10 (25.0)
Chemotherapy	
No	8 (20.0)
Yes	32 (80.0)

^a^According to the 7th American Joint Committee on Cancer (AJCC) staging system. WHO, World Health Organization.

### Treatment

All patients were fixed with a 5-point thermoplastic mask (Civco Medical Solutions, Kolona, IA, USA), which may also position the patients' shoulders. Target volumes were delineated according to the International Commission on Radiation Units and Measurements (ICRU) reports 50 and 62. The PTVs and planning organs at risk volume (PRVs) were generated by the addition of a 3-mm margin to both the delineated target volume and corresponding structures (such as the spinal cord, brainstem, and optic nerve pathway). The prescribed dose was 70 Gy to the PTV of the gross tumor volume of the primary site (GTV-P), 64–66 Gy to the PTV of the nodal gross tumor volume (GTV-N), 60 Gy to the PTV of the clinical target volume-1 (CTV-1; high-risk regions), and 56 Gy to the PTV of the CTV-2 (low-risk regions) and the CTV-N (nodal regions in the neck) in 33 fractions. The PTVs of GTV, CTV1, and CTV2 were named PTV_7000, PTV_6000, and PTV_5600, respectively. All patients were treated with 1 fraction daily, 5 days a week. Neoadjuvant and adjuvant chemotherapy and concomitant chemotherapy with a platinum-based protocol were recommended for patients with stage III to IVB NPC.

### PTV margins

To study the dosimetric impact of margin reductions on the IMRT plan, two additional PTVs with pre-correction and post-correction PTV margins were generated for each patient. We obtained the pre-correction and post-correction PTV margins through our preliminary study. The process was as follows.

First, after conventional positioning by aligning the in-room lasers with the marks drawn on the masks, we obtained the first CBCT images. Second, the acquired CBCT images were registered to the planning CT scan by using automatic bone matching (Elekta XVI software, Elekta, Crawley, UK) to obtain the translational errors of target on the medial-lateral (ML), superior-inferior (SI), and anterior-posterior (AP) directions. If a translational error was greater than 2 mm in any direction, setup corrections were made by adjusting the patient’s position through automatically shifting the treatment couch in all ML, SI, and AP directions. Third, after setup correction, a second CBCT scan was performed and registered to the planning CT scan, thereby obtaining the residual setup error. Finally, the fraction radiation therapy was performed. The difference between the pre-correction CBCT image and planning CT was pre-correction setup error, whereas the difference between the post-correction CBCT image and planning CT image was post-correction setup error. We followed the geometric margin formula developed by van Herk *et al*. [13] to calculate the pre-correction and post-correction PTV margins with pre-correction and post-correction errors, respectively.

The pre-correction and post-correction systematic setup uncertainty, random setup uncertainty, and PTV margins, obtained during preliminary research in our center, are shown in Table 2. Post-correction margins were clearly smaller than the pre-correction margins in the 3D direction, and the margins decreased by 48% − 63% after online correction in the three directions.

**Table 2:** **Pre-correction and post-correction systematic setup uncertainty, random setup uncertainty, and planning target volume (PTV) margins in three-dimensional directions**

**Variable**	**Pre-correction**			**Post-correction**		
	**ML**	**SI**	**AP**	**ML**	**SI**	**AP**
Σ^a^ (mm)	1.4	1.0	1.0	0.4	0.5	0.4
σ^b^ (mm)	0.8	0.8	0.7	0.6	0.7	0.7
PTV (mm)	4.0	3.1	3.1	1.5	1.7	1.6

^a^Systematic setup uncertainty. ^b^Random setup uncertainty. ML, medial-lateral; SI, superior-inferior; AP, anterior-posterior.

### Plan re-optimization

Optimization was performed by using the Monaco treatment planning system (version 3.1, Elekta Medical Systems, Crawley, UK) and doses were calculated with the Monte Carlo algorithm [14]. Both plans with pre-correction and post-correction margins were generated for an Elekta Synergy linear accelerator (Elekta, Crawley, UK) using 6-MV photons. A standard constraint set referring to RTOG0615 was used for optimization and evaluation. The aim was to achieve 95% of any PTV at or above the prescription dose, 99% of any PTV at or above 93% of the PTV dose, no more than 20% of the PTV_7000 at or above 77 Gy (that is, 110% of the PTV_7000 dose), and no more than 5% of any PTV_7000 at or above 80.5 Gy (that is, 115% of the PTV_7000 dose). For OARs, the most important objective was to keep maximum doses to the 1% of the PRV of the spinal cord (SpinalCord_PRV) below 50 Gy and to the 1% of the PRV of the brain stem (BrainStem_PRV) below 60 Gy. The second priority was to ensure that 50% of the parotid glands received a dose < 30 Gy (to be achieved in at least one gland). All targets were treated simultaneously by using the simultaneous integrated boost (SIB) technique.

### Plan comparison

A quantitative comparison of plans was performed by using the standard dose-volume histogram (DVH). The DVH parameters for target comparisons refer to ICRU 83. In both plans, the median dose, D_2%_, D_95%_, and D_98%_ (doses that cover 2%, 95%, and 98% of the PTV, respectively) were recorded for each PTV. Values of D_98%_ and D_2%_ were defined as metrics for minimum and maximum doses, respectively. A further measurement of dose homogeneity was expressed by the homogeneity index (HI), which was (D_2%_–D_98%_)/D_50%_, and a high HI indicates poor homogeneity.

For OARs, the analysis included the maximum dose, the mean dose, and a set of appropriate V_X_ (percentage volume receiving less/more than X Gy) and D_Y_ (dose received by Y volume) values.

### Statistical analysis

All analyses were performed using SPSS software, version 16.0 (SPSS, Chicago, IL, USA). Paired *t* tests were used to compare the dosimetry of plans with pre-correction margins and those with post-correction margins. Pearson’s correlation analysis was used to evaluate associations between differences in DVH parameters for both PTV scenarios and tumor volume, and a one-way analysis of variance (ANOVA) was used to analyze differences in dosimetric improvements with various tumor stages. Two-tailed *P* values of < 0.05 were considered significant.

## Results

### Target coverage

The volumes of PTV with post-correction margins were greater than those of PTV with pre-correction margins (Table 3). Irrespective of margin reductions, all plans met the planning goals for target coverage (Table 4). Dosimetric differences in targets that resulted from margin reductions are summarized in Table 5. In general, significant improvements in target coverage were observed following margin reductions. Regarding HI and D_2%_, plans with post-correction margins showed larger values compared with plans with pre-correction margins at all dose levels (*P* < 0.05). The D_98%_ of PTV_7000 was larger for plans with post-correction margins than that for plans with pre-correction margins (*P* < 0.05), but there were no significant differences in PTV_6000 and PTV_5600 (*P* = 0.805 and 0.990, respectively).

**Table 3:** **The tumor volume for targets in 40 NPC patients**

**Target**		**Mean (mL)**	**SD (mL)**	**Range (mL)**
GTV		35.44	29.02	1.17–119.70
CTV1		112.04	50.42	21.69–239.26
CTV2		493.20	134.24	75.24–792.59
PTV_7000	Pre-correction	68.20	42.78	4.51–186.32
	Post-correction	54.65	36.02	3.09–161.97
PTV_6000	Pre-correction	166.75	66.79	40.70–333.05
	Post-correction	147.16	61.98	33.99–299.74
PTV_5600	Pre-correction	745.13	155.04	364.14–1,108.09
	Post-correction	650.69	141.54	311.18–988.26

GTV, gross tumor volume; CTV, clinical target volume; PTV_7000, the PTV of gross tumor volume; PTV_6000, the PTV of high-risk regions; PTV_5600, the PTV of low-risk regions; SD, standard deviation.

**Table 4:** **Comparison of NPC patients not fulfilling dose-volume histogram constraints for plans with pre-correction margins and for plans with post-correction margins**

**Organ**		**Objective**	**Plan with pre-correction margins [** ***n*** **(%)]**	**Plan with post-correction margins [** ***n*** **(%)]**	**Reduction (%)**
Targets	Any PTV	V_93%_ ≥ 99%	0 (0)	0 (0)	0.0
		V_100%_ ≥ 95%	0 (0)	0 (0)	0.0
	PTV_7000	V_77Gy_ ≤ 20%	0 (0)	0 (0)	0.0
		V_80.5Gy_ ≤ 5%	0 (0)	0 (0)	0.0
OARs	BrainStem	D_max_ ≤ 54 Gy	23 (57.5)	22 (55.0)	4.3
	BrainStem_PRV	D_1%_ ≤ 60 Gy	12 (30.0)	8 (20.0)	33.3
	SpinalCord	D_max_ ≤ 45 Gy	0 (0)	0 (0)	0.0
	SpinalCord_PRV	D_1%_ ≤ 50 Gy	0 (0)	0 (0)	0.0
	OpticNerve_L	D_max_ ≤ 50 Gy	21 (52.5)	19 (47.5)	9.5
	OpticNerve_L_PRV	D_1%_ ≤ 54 Gy	21 (52.5)	17 (42.5)	19.0
	OpticNerve_R	D_max_ ≤ 50 Gy	22 (55.0)	19 (47.5)	13.6
	OpticNerve_R_PRV	D_1%_ ≤ 54 Gy	21 (52.5)	19 (47.5)	9.5
	Chiasm	D_max_ ≤ 50 Gy	26 (65.0)	26 (65.0)	0.0
	Chiasm_PRV	D_1%_ ≤ 54 Gy	27 (67.5)	27 (67.5)	0.0
	Lens	D_max_ < 25 Gy	0 (0)	0 (0)	0.0
	Parotids	V_20Gy_ > 20 cc (both glands)	40 (100)	40 (100)	0.0
		D_50%_ < 30 Gy (at least one gland)	28 (70.0)	19 (47.5)	32.1
	Parotid_L	D_mean_ < 26 Gy	40 (100)	40 (100)	0.0
	Parotid_R	D_mean_ < 26 Gy	40 (100)	40 (100)	0.0
	TemporalLobe_L	D_max_ ≤ 60 Gy	39 (97.5)	38 (95.0)	2.6
		D_1%_ ≤ 65 Gy	15 (37.5)	12 (30.0)	20.0
	TemporalLobe_R	D_max_ ≤ 60 Gy	39 (97.5)	38 (95.0)	2.6
		D_1%_ ≤ 65 Gy	16 (40.0)	13 (32.5)	18.8
	Mandible/TM joint	D_1cc_ ≤ 75 Gy	0 (0)	0 (0)	0.0
	Ear_Inner_L	V_55Gy_ ≤ 5%	30 (75.0)	27 (67.5)	10.0
	Ear_Inner_R	V_55Gy_ ≤ 5%	34 (85.0)	26 (65.0)	23.5
	Larynx	D_mean_ < 45 Gy	31 (77.5)	28 (70.0)	9.7

OAR, organ at risk; PTV, planning target volume; PTV_7000, the PTV of gross tumor volume; PRV, planning risk volume; L, left; R, right; TM, temporomandibular; V_93%_, percentage volume receiving at least 93% of prescribed dose; V_100%_, percentage volume receiving at least 100% of prescribed dose; V_77Gy_, percentage volume receiving at least 77 Gy; V_80.5Gy_, percentage volume receiving at least 80.5 Gy; D_1%_, dose received by 1% of the volume; D_max_, maximum dose; V_20Gy_, volume receiving less than 20 Gy; cc, cubic centimeter; D_50%_, dose received by 50% of the volume; D_mean_, mean dose; D_1cc_, dose received by 1 cc volume; V_55Gy_, percentage volume receiving at least 55 Gy.

**Table 5:** **Dosimetric comparison of plans with pre-correction margins and plans with post-correction margins for the PTVs in 40 NPC patients**

**Target**	**Dose index**	**Plan with pre-correction margins**	**Plan with post-correction margins**	**Difference**	***P*** **value**
PTV_7000	D_2%_ (Gy)	76.2 ± 0.84	75.97 ± 0.79	0.28 ± 0.57	0.003
	D_50%_ (Gy)	73.79 ± 0.43	73.59 ± 0.38	0.19 ± 0.32	<0.001
	D_95%_ (Gy)	71.05 ± 0.32	71.06 ± 0.26	−0.01 ± 0.16	0.738
	D_98%_ (Gy)	69.53 ± 1.34	69.80 ± 1.01	−0.27 ± 0.44	<0.001
	HI	0.09 ± 0.03	0.08 ± 0.02	0.01 ± 0.01	<0.001
PTV_6000	D_2%_ (Gy)	75.77 ± 0.85	75.44 ± 0.80	0.33 ± 0.48	<0.001
	D_50%_ (Gy)	71.18 ± 1.29	70.79 ± 1.22	0.39 ± 0.30	<0.001
	D_95%_ (Gy)	64.00 ± 1.06	63.85 ± 1.06	0.16 ± 0.44	0.030
	D_98%_ (Gy)	62.33 ± 1.04	62.34 ± 1.07	−0.02 ± 0.45	0.805
	HI	0.19 ± 0.02	0.18 ± 0.02	0.01 ± 0.01	0.010
PTV_5600	D_2%_ (Gy)	74.60 ± 1.04	74.33 ± 1.06	0.27 ± 0.38	<0.001
	D_50%_ (Gy)	62.58 ± 0.77	62.41 ± 0.83	0.17 ± 0.40	0.009
	D_95%_ (Gy)	57.81 ± 0.56	57.72 ± 0.54	0.09 ± 0.22	0.018
	D_98%_ (Gy)	55.69 ± 1.06	55.70 ± 1.08	−0.01 ± 0.35	0.990
	HI	0.30 ± 0.03	0.29 ± 0.03	0.01 ± 0.01	0.002

All data are presented as mean ± SD of 40 patients. HI, homogeneity index; D_2%_, dose received by 2% of the volume; D_50%_, dose received by 50% of the volume; D_95%_, dose received by 95% of the volume; D_98%_, dose received by 98% of the volume. Other abbreviations as in Tables 3 and 4.

### OARs

Depending on the margins used, dose distributions of the OARs that did not comply with the planning objectives are shown in Table 4. Only the spinal cord, lens, mandible, and temporomandibular (TM) joint dose distributions complied with the planning objectives for all patients, irrespective of the margin strategy. However, with margin reductions, there was a 33.3% reduction in the number of patients who did not fulfill the criteria of D_1%_ <60 Gy for BrainStem_PRV and a 32.1% reduction in the planning objective of D_50%_ <30 Gy for the parotids. Dose distribution improvements from margin reductions for the brainstem and the parotids were consistently better than those for other OARs.

**Figure 1:**
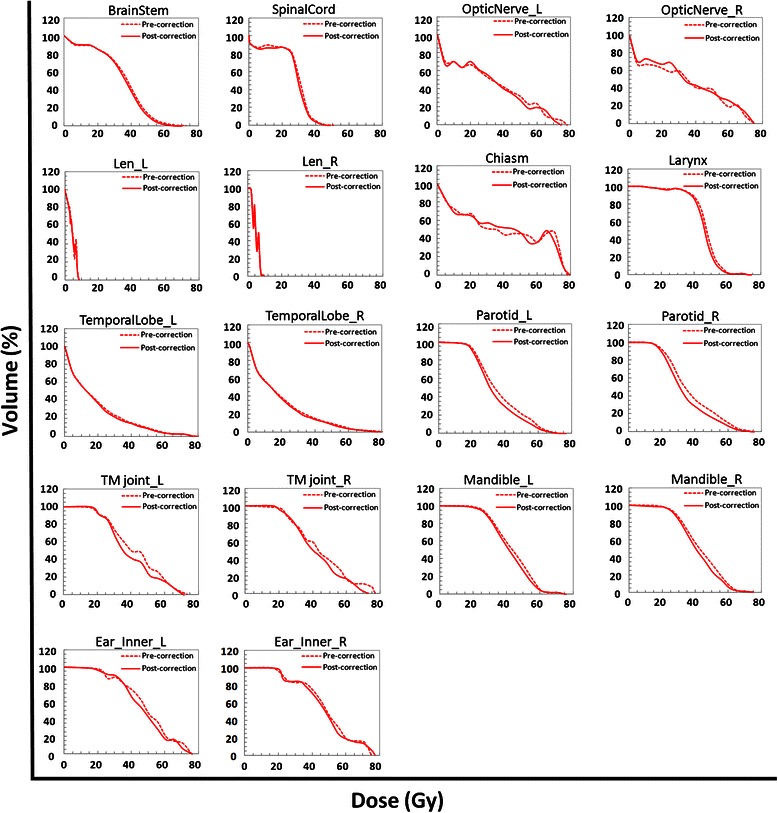
Average dose volume histograms of plans with pre-correction margins and plans with post-correction margins for all 40 patients with nasopharyngeal carcinoma. Each picture is based on the average dose volume histogram to an organ at risk. L, left; R, right; TM, temporomandibular.

A favorable dosimetric impact of margin reductions on OARs was observed (Table 6). The average DVH for all the OARs, comparing the two different margin strategies for the entire patient cohort, is shown in Figure 1. Significant differences in dosimetric parameters between the two margin strategies were observed for all OARs (*P* < 0.05), apart from the PRV of the right optic nerve (OpticNerve_R_PRV), the PRV of the chiasm (Chaism_PRV), and the PRV of the lens. With margin reductions, the maximum doses to BrainStem_PRV, SpinalCord_PRV, and the PRV of the left optic nerve (OpticNerve_L_PRV) were decreased by 2.3%, 2.6%, and 1.7%, respectively, whereas the mean doses to the left parotid, right parotid, left inner ear, right inner ear, and larynx were decreased by 7.1%, 7.7%, 3.3%, 3.6%, and 2.3%, respectively. Additionally, the D_1%_ decreased at a rate of 1.5% and 2.2% in the left and right temporal lobes, respectively, and the dose received by 1 cubic centimeter volume (D_1cc_) reduced by 2.8%, 3.4%, 5.4%, and 3.7% in the left mandible, right mandible, left TM joint, and right TM joint, respectively. Sparing of the left and right parotids was consistently better than that observed with other OARs. Planning objectives for healthy tissue were not formalized numerically, but the strategy was to minimize the involvement of these tissues.

**Table 6:** **Dosimetric comparisons of plans with pre-correction margins and plans with post-correction margins for OARs in 40 NPC patients**

**Organ**	**Dose index**	**Plans with pre-correction margins**	**Plans with post-correction margins**	**Difference**	***P*** **value**
BrainStem_PRV	D_1%_ (Gy)	56.93 ± 5.48	55.62 ± 5.23	1.31 ± 1.08	<0.001
SpinalCord_PRV	D_1%_ (Gy)	37.95 ± 3.81	36.91 ± 3.45	1.04 ± 1.41	<0.001
OpticNerve_L_PRV	D_1%_ (Gy)	51.89 ± 15.01	50.97 ± 14.48	0.91 ± 2.20	0.012
OpticNerve_R_PRV	D_1%_ (Gy)	51.58 ± 16.49	50.90 ± 16.57	0.68 ± 2.81	0.134
Chiasm_PRV	D_1%_ (Gy)	58.84 ± 13.35	58.63 ± 12.90	0.21 ± 2.36	0.578
Lens_L	D_max_ (Gy)	5.85 ± 1.81	5.81 ± 1.82	0.04 ± 0.23	0.327
Lens_R	D_max_ (Gy)	5.88 ± 1.86	5.84 ± 1.84	0.04 ± 0.21	0.223
Parotid_L	D_mean_ (Gy)	37.31 ± 4.29	34.66 ± 4.17	2.65 ± 1.33	<0.001
	V_30Gy_ (%)	60.81 ± 15.83	53.35 ± 15.39	7.46 ± 7.18	<0.001
Parotid_R	D_mean_ (Gy)	37.31 ± 4.08	34.44 ± 4.20	2.87 ± 1.42	<0.001
	V_30Gy_ (%)	61.16 ± 15.25	52.27 ± 15.92	8.89 ± 8.51	<0.001
TemporalLobe_L	D_1%_ (Gy)	63.67 ± 6.94	62.73 ± 7.47	0.94 ± 2.17	0.009
TemporalLobe_R	D_1%_ (Gy)	64.58 ± 6.24	63.14 ± 6.35	1.44 ± 2.03	<0.001
Mandible_L	D_1cc_ (Gy)	60.24 ± 3.43	58.56 ± 4.36	1.67 ± 2.10	<0.001
Mandible_R	D_1cc_ (Gy)	60.28 ± 4.56	58.28 ± 5.18	2.00 ± 1.83	<0.001
TMjoint_L	D_1cc_ (Gy)	44.67 ± 10.59	42.16 ± 10.79	2.51 ± 4.24	0.001
TMjoint_R	D_1cc_ (Gy)	45.46 ± 12.14	43.40 ± 10.99	2.06 ± 3.25	<0.001
Ear_Inner_L	D_mean_ (Gy)	49.36 ± 7.98	47.69 ± 7.67	1.67 ± 1.61	<0.001
Ear_Inner_R	D_mean_ (Gy)	49.86 ± 8.42	48.00 ± 8.17	1.87 ± 2.22	<0.001
Larynx	D_mean_ (Gy)	47.09 ± 2.57	45.98 ± 2.52	1.11 ± 1.46	<0.001

All data are presented as mean ± SD of 40 patients. V_30Gy_, percentage volume receiving at least 30 Gy. Other abbreviations as in Tables 3 and 4.

Our results showed that the DVH parameters for each OAR didn’t decrease significantly with margin reduction when dividing the patients into different groups according to the tumor size (*P* > 0.05). To analyze the association between the DVH parameters and T category, we divided the patients into 4 groups according to the T category. Our results showed that, only the percentage volume receiving at least 60 Gy (V_60Gy_) of the brainstem significantly decreased with shrank margin in patients with T4 lesions (*P* = 0.001). However, the reductions in the maximum doses to the brainstem, optic nerve, chiasm, and right lens, the mean doses to the left parotid and ear, and D_1cc_ to the right TM joint were more apparent in patients with T4 lesions, but no significant difference was observed among the 4 groups.

## Discussion

Many reports have demonstrated that the dose distribution can be improved by minimizing PTV margin [6-8]. According to our knowledge, this study is the first to report the dosimetric effect of PTV margin reduction during the treatment of NPC patients and demonstrate improvements both in OAR sparing and target coverage.

Recent studies have described PTV margin reductions for multiple correction protocols in radiotherapy for head and neck cancer. Den *et al.* [11] and Wang *et al.* [12] reported that CBCT online correction with 2-mm tolerance resulted in 40%–60% and 51%–68% reduction in PTV margins, respectively. Velec *et al.* [15] concluded that CBCT facilitated a 13%–39% reduction in PTV margins with a combination of online correction with 3-mm tolerance and offline correction. Rates of margin reductions in our study are similar to those reported by Den *et al.* [11] and Wang *et al.* [12] but greater than those reported by Velec *et al.* [15]. This difference may be due to the various correction thresholds and correction protocols used in these studies. Generally, a smaller correction threshold was associated with a higher rate of margin reductions. Additionally, online correction can reduce both systematic error and random error, whereas offline correction can only reduce systematic error [16,17]. Therefore, the rate of margin reduction can be increased with online correction.

Improvements in dose distributions with margin reductions have been reported for many tumors, but not for NPC. Grills *et al*. [7] reduced the margins surrounding lung tumors from 9–13 mm to 2–4 mm with online correction using CBCT, and this reduction in margins resulted in 23% − 32% dose reduction in average lung dose, 19% − 27% dose reduction in maximum spinal cord dose, and 10% − 22% dose reduction in maximum esophageal dose. In our study, the reduced margins decreased the dose to OARs with values ranging from 1.5% to 7.7% and provided better target coverage. Compared with the result reported for lung cancer, the margin reduction for NPC was smaller, and the decreases of OAR doses caused by margin reduction were less. This finding may have resulted from higher reproducibility and stability of the patient position for NPC than those for thoracic tumors. Our results demonstrate the dosimetric advantage of reduced margins using CBCT online correction for NPC.

Additionally, in our study, planning objectives of the brainstem and parotids were not fulfilled in 57.5% and 70.0% of patients, respectively, for plans with pre-correction margins. However, among those patients who did not have a D_1%_ less than 60 Gy for BrainStem_PRV, a 33.3% margin reduction was observed. Additionally, there was a 32.1% reduction in patients who did not fulfill the planning objective of D_50%_ < 30 Gy for the parotids. The pass rates of the radiotherapy plan from margin reductions for the brainstem and parotids were consistently higher than those observed for other OARs. This observation may be explained by the close proximity of the tumors to both the brainstem and parotids, and margin reductions result in a decrease in the overlap volume between the two OARs and the tumors, thereby decreasing the volume of the OARs irradiated at high dose levels. Therefore, margin reductions can achieve the planning objectives of the OARs, thereby improving the acceptance and tolerance rates of the radiotherapy plan.

Sparing of the parotid glands was most obvious, with a decrease in the mean dose up to 7.7%. This result suggests that patients could have a dose decrease if planned with small PTV margins and treated with the post-correction position, thereby enhancing treatment efficacy. Additionally, with CBCT online correction, the margins decreased to 2.5 mm, 1.4 mm, and 1.5 mm in the ML, SI, and AP directions, respectively. This reduction resulted in a decrease in mean doses of 2.65 Gy and 2.87 Gy to the left and right parotids, respectively. These results are similar to those reported by van Asselen *et al.* [18], who observed that the mean dose to the parotids increases linearly with increasing margins by approximately 1.3 Gy/mm. The occurrence of radiation adverse effects, which affect the quality of life, is related to the radiation dose. However, the present study involved only dosimetry. Further research is warranted to confirm whether the decrease of OAR dose observed in our study may reduce the incidence of adverse effects.

Our study showed that only the V_60Gy_ of the brainstem significantly decreased with shrank margin in patients with T4 lesions compared with those with T1–3 lesions (*P* < 0.01). This finding indicates that the high dose region of the brainstem was reduced significantly for patients with T4 lesions compared with those with other stage diseases. As we know, the value of V_60Gy_ is rarely greater than 0 in patients with T1 lesions, whereas it is rarely greater than 10% for patients with T4 lesions; it is larger for patients with T4 lesions than that for those with T1–3 lesions. Therefore, if there is a small reduction in PTV margins, the reduction of V_60Gy_ is more apparent for patients with T4 lesions than for patients with T1–3 lesions.

The reductions in the maximum dose to the brainstem, optic nerve, chiasm, and right lens, in the mean dose to the left parotid and ear, and in D_1cc_ to the right TM joint were more apparent for patients with T4 lesions compared with other groups of patients, but no significant difference was observed. A possible reason for this result was that the reductions in PTV margins were not large enough to achieve statistical significance among these groups. Another reason was the limited data used for the analysis: our study included only 40 patients, with 7–15 patients in each group.

Because many critical normal structures are in close proximity to the nasopharynx, it is important to protect OARs without compromising PTV coverage in NPC. First, accurate and consistent OAR delineation in NPC is critical for organ protection. Inaccurate delineation will mislead treatment planning, resulting in OAR overdose or inadequate target volume coverage. Sun *et al*. [19] found that different contouring methods can make the volume and dosimetric parameters of organs significantly different. Second, shrunken targets contributed to the protection of OARs. The common methods of narrowing the targets are reducing the PTV margins by improving the placement accuracy or modifying the target during the course of therapy. Furthermore, the sufficient sparing of critical normal structures in NPC patients could be achieved by the use of IMRT. As we know, IMRT offers superior dose conformity to tumor targets with relative sparing of critical organs. Results from retrospective and prospective studies have confirmed the efficacy of IMRT on disease control as well as the benefit in OAR sparing. Finally, appropriate dose limit parameters to OARs also contribute to sparing of critical structures. There are various ways to protect the OARs, and the most suitable method to decrease the radiation dose to OARs should be adopted.

## Conclusion

This study is the first to quantify the magnitude of sparing of OARs due to margin reductions with CBCT online correction in IMRT for the management of NPC. Our results confirmed improvements both in OAR sparing and target coverage, and the parotid gland benefited most from the online imaging-guided approach. However, prospective randomized trials should be performed to confirm whether the dosimetric benefit observed in our study can be translated into clinical benefit.
